# Supplementary material for: Negative social ties as emerging risk factors for accelerated aging, inflammation, and multimorbidity
Source: Proc Natl Acad Sci U S A. 2026 Feb 18;123(8):e2515331123. doi: 10.1073/pnas.2515331123 (PMC12933095; doi:10.1073/pnas.2515331123)
Supplement: Supplementary file 1 — Appendix 01 (PDF) [file pnas.2515331123.sapp.pdf]

PNAS

# Supporting Information for

Negative Social Ties as Emerging Risk Factors for Accelerated Aging, Inflammation, and Multimorbidity

Authors: Byungkyu Lee*, Gabriele Ciciurkaite, Siyun Peng, Colter Mitchell, Brea L. Perry*

*Corresponding authors: Byungkyu Lee (bklee@nyu.edu) and Brea Perry (blperry@iu.edu)

**This PDF file includes:**

Figure S1
Tables S1 to S15

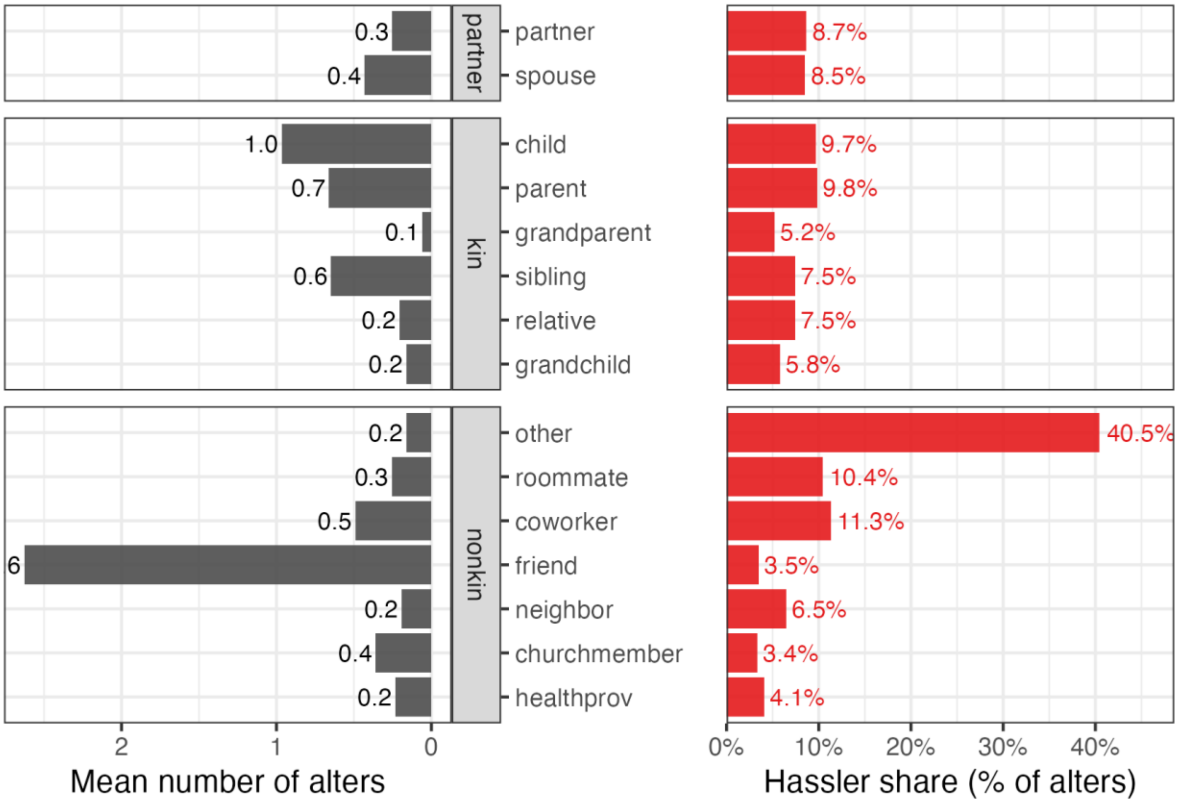


**Figure S1. The mean number of alters by relationship type and the proportion of hasslers in each relationship category.** Mean number of alters (left panels) and proportion of alters who are reported as "hasslers" (right panels) across partner, kin, and non-kin relationship categories. Bars show the number of each alter type (e.g., spouse, parent, coworker), and the percentage (i.e., the share of alters within each category who are identified as hasslers). All values reflect the average values across all respondents.

**Table S1. The descriptive statistics of individual characteristics and the key variables in the analytic sample**

| | Mean | SD | Min | Max | N (=2345) |
|---|---|---|---|---|---|
| Individual characteristics | | | | | |
| Age | 46.24 | 18.09 | 18 | 104 | |
| Gender: Female | 0.52 | 0.50 | 0 | 1 | |
| Race: White | 0.81 | 0.39 | 0 | 1 | |
| Race: Black | 0.10 | 0.30 | 0 | 1 | |
| Race: Hispanic | 0.09 | 0.29 | 0 | 1 | |
| Education: HS or less than HS | 0.30 | 0.46 | 0 | 1 | |
| Education: Some college | 0.38 | 0.49 | 0 | 1 | |
| Education: College or higher | 0.32 | 0.47 | 0 | 1 | |
| Marital Status: Never-married | 0.23 | 0.42 | 0 | 1 | |
| Marital Status: Married or cohabiting | 0.58 | 0.49 | 0 | 1 | |
| Marital Status: Widowed/Separated/Divorced | 0.19 | 0.40 | 0 | 1 | |
| Occupation: Professional/Managerial | 0.06 | 0.23 | 0 | 1 | |
| Occupation: Healthcare | 0.07 | 0.25 | 0 | 1 | |
| Occupation: Education & Social Services | 0.05 | 0.21 | 0 | 1 | |
| Occupation: Service | 0.06 | 0.23 | 0 | 1 | |
| Occupation: Sales & Office | 0.07 | 0.26 | 0 | 1 | |
| Occupation: Production & Transportation | 0.01 | 0.12 | 0 | 1 | |
| Occupation: Other | 0.01 | 0.09 | 0 | 1 | |
| Occupation: Not in labor force | 0.22 | 0.42 | 0 | 1 | |
| Occupation: Unemployed | 0.04 | 0.19 | 0 | 1 | |
| Occupation: Missing Industry | 0.42 | 0.49 | 0 | 1 | |
| Smoking: Never smoker | 0.55 | 0.50 | 0 | 1 | 2341 |
| Smoking: Ever smoker | 0.22 | 0.42 | 0 | 1 | 2341 |
| Smoking: Current smoker | 0.06 | 0.24 | 0 | 1 | 2341 |
| Smoking: Daily smoker | 0.17 | 0.37 | 0 | 1 | 2341 |
| % Leukocyte cells | 0.71 | 0.19 | 0 | 0.98 | |
| Batch Number (ref = 8615) | 0.16 | 0.36 | 0 | 1 | |
| Batch 8732 | 0.18 | 0.39 | 0 | 1 | |
| Batch 9054 | 0.24 | 0.43 | 0 | 1 | |
| Batch 9109 | 0.22 | 0.41 | 0 | 1 | |
| Batch 9213 | 0.15 | 0.36 | 0 | 1 | |
| Batch 11277 | 0.01 | 0.12 | 0 | 1 | |
| Batch 13762 | 0.04 | 0.20 | 0 | 1 | |
| Interview during COVID-19 | 0.39 | 0.49 | 0 | 1 | |
| Has Health Insurance | 0.92 | 0.27 | 0 | 1 | 2339 |
| Adverse childhood experiences (count) | 2.37 | 2.66 | 0 | 12 | |

| | | | | | |
|---|---|---|---|---|---|
| How important are you to others (binary) | 0.68 | 0.47 | 0 | 1 | 2299 |
| How much do others depend on you (binary) | 0.65 | 0.48 | 0 | 1 | 2336 |
| <u>Independent variables</u> | 5.30 | 2.95 | 0 | 25 | |
| Network Size: All | 0.42 | 0.89 | 0 | 8 | |
| Network Size: Negative ties | 0.08 | 0.16 | 0 | 1 | |
| % Hassler | 0.84 | 0.65 | 0 | 3 | |
| Mean Hassle Frequency | 0.06 | 0.23 | 0 | 2 | |
| | 0.22 | 0.68 | 0 | 7 | |
| | 0.24 | 0.69 | 0 | 8 | |
| | 0.06 | 0.23 | 0 | 1 | |
| | 0.14 | 0.35 | 0 | 1 | |
| | 0.17 | 0.37 | 0 | 1 | |
| | 2.37 | 2.66 | 0 | 12 | |
| <u>Outcome variables</u> | | | | | |
| AgeAccelGrim2 | -0.73 | 7.01 | -23.00 | 47.37 | |
| PACE | 1.19 | 0.19 | 0.73 | 1.93 | |
| Self-reported General Health | 2.69 | 0.99 | 1 | 5 | 2343 |
| Self-reported Mental Health | 2.55 | 1.06 | 1 | 5 | 2342 |
| Self-reported Physical Health | 2.83 | 1.03 | 1 | 5 | 2343 |
| Epigenetic inflammation score | 0.06 | 0.03 | 0.00 | 0.17 | |
| Charlson co-morbidity index (past 3yr) | 0.30 | 0.92 | 0 | 8 | 2054 |
| Any medical visit (past 3yr) | 0.75 | 0.43 | 0 | 1 | 2054 |
| Multi-morbidity index | 1.09 | 1.30 | 0 | 9 | |
| Anxiety Severity | 18.81 | 18.22 | 0 | 100 | 2288 |
| Depression Severity | 29.25 | 19.04 | 0 | 100 | 2288 |
| Waist to Hip Ratio | 0.91 | 0.10 | 0.40 | 1.29 | 1911 |
| BMI | 30.64 | 8.30 | 7.9 | 100.3 | 1967 |
| Obese | 0.48 | 0.50 | 0 | 1 | 1967 |
| Height | 169.44 | 11.29 | 105 | 199.5 | 1983 |

Note. Survey weights were applied to adjust the descriptive statistics. The sample size is 2,345 when the sample size column is missing.

**Table S2. The distribution of network size and the proportion of hasslers**

| Network Size | Freq | Percent | % Hasslers |
|---|---|---|---|
| 0 | 35 | 1.3 | |
| 1 | 97 | 3.6 | 7.2 |
| 2 | 232 | 8.6 | 6.9 |
| 3 | 415 | 15.5 | 6.8 |
| 4 | 461 | 17.2 | 9.4 |
| 5 | 404 | 15 | 8.3 |
| 6 | 343 | 12.8 | 7.8 |
| 7 | 247 | 9.2 | 8.8 |
| 8 | 139 | 5.2 | 9.5 |
| 9 | 80 | 3 | 7.4 |
| 10+ | 232 | 8.6 | 8.4 |
| Total | 2685 | 100 | |
| mean | 5.07 | | 8.1 |
| sd | 2.46 | | 15.9 |

**Table S3. The distribution of the size of negative ties.**

| | Negative ties | |
|---|---|---|
| **N alters** | **Freq** | **%** |
| 0 | 1912 | 71.2 |
| 1 | 522 | 19.4 |
| 2 | 170 | 6.3 |
| 3 | 43 | 1.6 |
| 4 | 21 | 0.8 |
| 5 | 10 | 0.4 |
| 6+ | 7 | 0.3 |
| Total | 2685 | 100 |
| mean | 0.43 | |
| sd | 0.85 | |

**Table S4. Regression results from the zero-inflated Poisson regression model for the presence and the number of hasslers in ego networks.**

| | Model Coefficients | | Average Marginal Effects | |
|---|---|---|---|---|
| Outcome | Zero Hasslers | Count of Hasslers | Zero Hasslers | Count of Hasslers |
| Age (SD) | 0.411 | 0.0604 | 0.0258+ | -0.0305 |
| | (0.249) | (0.108) | (0.0135) | (0.0304) |
| Network size (SD) | -0.235 | 0.448** | -0.0947** | 0.221** |
| | (0.230) | (0.0591) | (0.0126) | (0.0222) |
| Self-reported general health (SD) | -0.175 | -0.268** | 0.0292* | -0.0891** |
| | (0.191) | (0.0959) | (0.0120) | (0.0265) |
| Lifetime Multimorbidity Index (SD) | -0.488 | -0.149 | -0.0178 | 0.00386 |
| | (0.360) | (0.0984) | (0.0221) | (0.0257) |
| ACEs score (SD) | -0.701** | 0.0743 | -0.0734** | 0.127** |
| | (0.179) | (0.0674) | (0.0118) | (0.0212) |
| Health Insurance | -1.129 | -0.373 | -0.0364 | -0.00344 |
| | (0.793) | (0.301) | (0.0311) | (0.0769) |
| Survey during COVID-19 | -0.195 | -0.295 | 0.0320 | -0.0979* |
| | (0.862) | (0.312) | (0.0341) | (0.0469) |
| How much do others depend on you | -0.693+ | 0.0341 | -0.0659* | 0.109+ |
| | (0.405) | (0.141) | (0.0294) | (0.0561) |
| How important are you to others | 0.957 | -0.00179 | 0.0836** | -0.131** |
| | (0.606) | (0.186) | (0.0267) | (0.0488) |
| Education (ref = College or higher) | | | | |
| HS or less than HS | 0.308 | 0.215 | -0.0120 | 0.0523 |
| | (0.546) | (0.216) | (0.0339) | (0.0668) |
| Some college | 0.777 | 0.254 | 0.0251 | -0.00107 |
| | (0.498) | (0.240) | (0.0298) | (0.0625) |
| Race (ref = White) | | | | |
| Black | 0.235 | 0.218 | -0.0138 | 0.0604 |
| | (0.766) | (0.305) | (0.0436) | (0.0848) |
| Other | -0.155 | 0.100 | -0.0308 | 0.0655 |
| | (0.881) | (0.217) | (0.0801) | (0.145) |
| Female (vs male) | -0.870 | -0.0781 | -0.0646* | 0.0886+ |
| | (0.616) | (0.202) | (0.0272) | (0.0477) |
| Marital Status (ref = Never-married) | | | | |
| Married or living with a partner | 0.123 | 0.249 | -0.0299 | 0.0860 |
| | (0.724) | (0.296) | (0.0341) | (0.0724) |
| Widowed/Divorced/Separated | -0.269 | -0.00239 | -0.0207 | 0.0305 |
| | (0.817) | (0.326) | (0.0332) | (0.0616) |

| | | | | |
|---|---|---|---|---|
| Occupation (ref = Unemployed) | 0.260 | -0.199 | 0.0637 | -0.170 |
| Professional/Managerial | (1.836) | (0.550) | (0.138) | (0.212) |
| | 0.491 | -0.556 | 0.141 | -0.354* |
| Healthcare | (1.490) | (0.365) | (0.119) | (0.177) |
| | 0.492 | -0.789 | 0.173 | -0.423* |
| Education & Social Services | (1.662) | (0.491) | (0.125) | (0.197) |
| | 0.867 | -0.143 | 0.119 | -0.253 |
| Service | (1.620) | (0.402) | (0.136) | (0.209) |
| | 0.437 | -0.401 | 0.113 | -0.290 |
| Sales & Office | (1.539) | (0.415) | (0.124) | (0.196) |
| | -21.33** | -1.087** | 0.0560 | -0.295 |
| Production & Transportation | (4.784) | (0.387) | (0.141) | (0.216) |
| | -0.148 | -0.491 | 0.0763 | -0.248 |
| Other | (1.480) | (0.418) | (0.124) | (0.208) |
| | -0.715 | -0.744* | 0.0823 | -0.293 |
| Not in labor force | (1.282) | (0.291) | (0.109) | (0.183) |
| | | | | |
| Missing Industry | 0.271 | -0.390 | 0.0962 | -0.261 |
| | (1.544) | (0.415) | (0.120) | (0.184) |
| Smoking status (ref = never smoker) | | | | |
| ever smoker | 0.226 | -0.0435 | 0.0260 | -0.0454 |
| | (0.541) | (0.178) | (0.0342) | (0.0564) |
| current smoker | 1.122 | 0.341 | 0.0559 | -0.0372 |
| | (0.786) | (0.415) | (0.0445) | (0.112) |
| daily smoker | -0.380 | 0.224 | -0.0751* | 0.162+ |
| | (0.652) | (0.296) | (0.0342) | (0.0876) |
| Constant | 0.471 | 0.0860 | | |
| | (1.483) | (0.448) | | |
| Observations | 2596 | | 2596 | 2596 |

Note. We estimate a Zero-inflated Poisson regression model using Stata's svy: zip command to account for the complex survey design. We report both regression coefficients and average marginal effects for the probability of zero hasslers (Pr(0)) and for the expected count of hasslers. Standard errors are shown in parentheses (+ p < 0.1, * p < 0.05, ** p < 0.01).

**Table S5. OLS regression models predicting two epigenetic aging clocks.**

| Outcome | PACE | | | GrimAge2 | | |
|---|---|---|---|---|---|---|
| | Model1 | Model2 | Model3 | Model4 | Model5 | Model6 |
| Batch Number (ref = 8615) | | | | | | |
| 8732 | -0.0286** | -0.0296** | -0.0291** | -0.530 | -0.580 | -0.538 |
| | (0.00807) | (0.00809) | (0.00786) | (0.490) | (0.485) | (0.492) |
| 9054 | -0.0290** | -0.0286** | -0.0270* | 0.129 | 0.145 | 0.169 |
| | (0.0107) | (0.0106) | (0.0105) | (0.356) | (0.362) | (0.358) |
| 9109 | -0.00798 | -0.00820 | -0.00766 | 0.0326 | 0.0246 | 0.0369 |
| | (0.0106) | (0.0108) | (0.0103) | (0.466) | (0.468) | (0.457) |
| 9213 | -0.0529** | -0.0535** | -0.0515** | -0.433 | -0.464 | -0.403 |
| | (0.0121) | (0.0123) | (0.0119) | (0.477) | (0.492) | (0.482) |
| 11277 | 0.0393 | 0.0383 | 0.0387 | 0.354 | 0.306 | 0.361 |
| | (0.0433) | (0.0434) | (0.0436) | (0.838) | (0.872) | (0.806) |
| 13762 | -0.0479* | -0.0490* | -0.0467* | -0.629 | -0.692 | -0.613 |
| | (0.0211) | (0.0210) | (0.0211) | (0.968) | (0.961) | (0.971) |
| % Leukocyte cells | -0.679** | -0.683** | -0.684** | -26.40** | -26.60** | -26.49** |
| | (0.0360) | (0.0357) | (0.0365) | (0.870) | (0.853) | (0.873) |
| Age | 0.000769** | 0.000764** | 0.000806** | -0.0482** | -0.0487** | -0.0474** |
| | (0.000271) | (0.000273) | (0.000272) | (0.00866) | (0.00881) | (0.00879) |
| Race (ref = White) | | | | | | |
| Black | 0.0830** | 0.0843** | 0.0843** | 2.434** | 2.503** | 2.454** |
| | (0.0119) | (0.0119) | (0.0119) | (0.535) | (0.535) | (0.525) |
| Other | 0.0531** | 0.0555** | 0.0542** | 1.380** | 1.509* | 1.360** |
| | (0.00923) | (0.00999) | (0.00950) | (0.505) | (0.575) | (0.491) |
| FEMALE | 0.0306** | 0.0305** | 0.0304** | -0.254 | -0.248 | -0.255 |
| | (0.00615) | (0.00610) | (0.00602) | (0.231) | (0.233) | (0.232) |
| Education (ref = HS or less than HS) | | | | | | |
| Some college | -0.0234+ | -0.0240+ | -0.0233+ | -0.929* | -0.966* | -0.916* |
| | (0.0121) | (0.0120) | (0.0123) | (0.442) | (0.440) | (0.448) |
| College or higher | -0.0590** | -0.0606** | -0.0590** | -2.772** | -2.862** | -2.763** |
| | (0.00963) | (0.00958) | (0.00977) | (0.445) | (0.439) | (0.453) |

| | | | | | | |
|---|---|---|---|---|---|---|
| Martial Status (ref = Never-married) | | | | | | |
| Married or living with a partner | -0.00560 | -0.00573 | -0.00442 | 0.590 | 0.592 | 0.607 |
| | (0.0126) | (0.0129) | (0.0126) | (0.366) | (0.374) | (0.364) |
| Widowed/Divorced/Separated | 0.0196 | 0.0190 | 0.0187 | 1.860** | 1.834** | 1.841** |
| | (0.0125) | (0.0125) | (0.0123) | (0.460) | (0.466) | (0.466) |
| Number of hasslers | 0.0152** | | | 0.784** | | |
| | (0.00384) | | | (0.189) | | |
| Presence of any hassler | | 0.0258** | | | 1.237** | |
| | | (0.00709) | | | (0.296) | |
| N Hasslers (ref = 0) | | | | | | |
| 1 | | | 0.0108 | | | 0.679* |
| | | | (0.00782) | | | (0.290) |
| 2 | | | 0.0682** | | | 2.324** |
| | | | (0.0157) | | | (0.369) |
| 3 | | | 0.0426 | | | 1.774+ |
| | | | (0.0331) | | | (0.950) |
| 4 | | | 0.0966+ | | | 4.570** |
| | | | (0.0534) | | | (1.144) |
| 5+ | | | -0.0131 | | | 2.642 |
| | | | (0.0223) | | | (1.900) |
| Constant | 1.646** | 1.649** | 1.646** | 20.19** | 20.37** | 20.19** |
| | (0.0595) | (0.0592) | (0.0599) | (1.290) | (1.296) | (1.296) |
| Observations | 2345 | 2345 | 2345 | 2345 | 2345 | 2345 |

Note. All models control for network size dummies. Standard errors are in parentheses (+ $p < 0.1$, * $p < 0.05$, ** $p < 0.01$).

**Table S6. Results from the alternative specification of negative ties.**

| | PACE | | | AgeAccelGrim2 | | |
|---|---|---|---|---|---|---|
| | Model1 | Model2 | Model3 | Model4 | Model5 | Model6 |
| % Negative Ties | 0.0796** | | | 4.040** | | |
| | (0.0203) | | | (1.037) | | |
| % Hasslers (Ref = 0%) | | | | | | |
| % Hasslers < 25% | | 0.0142 | | | 1.016** | |
| | | (0.00934) | | | (0.261) | |
| % Hasslers < 50% | | 0.0356** | | | 1.091* | |
| | | (0.0133) | | | (0.411) | |
| % Hasslers < 100% | | 0.0142 | | | 1.324 | |
| | | (0.0213) | | | (0.905) | |
| % Hasslers = 100% | | 0.0831* | | | 4.978** | |
| | | (0.0325) | | | (1.576) | |
| Avg Hassling Frequency | | | 0.0149* | | | 0.702** |
| | | | (0.00631) | | | (0.246) |
| Observations | 2345 | 2345 | 2345 | 2345 | 2345 | 2345 |
| Controls | | | | | | |
| Individual controls | V | V | V | V | V | V |
| Network size dummies | V | V | V | V | V | V |

Note. Model 1 and 4 = the proportion of hasslers; Model 2 and 5 = categorical measures of the proportion of hasslers; Model 3 and 6 = the mean hassling frequency. Coefficients represent the average marginal effects of negative social ties on biological aging outcomes, with standard errors in parentheses. Individual controls include age, gender, race/ethnicity, education, and marital status. Network size dummies account for differences in social network size, ensuring that the relative impact of negative ties is not confounded by absolute network size. Statistical significance is denoted as follows: + p < .10, * p < .05, ** p < .01 (two-tailed tests).

**Table S7. Associations between Negative Social Ties and Other Health Outcomes.**

| Outcome | General Health | Mental Health | Physical Health | Anxiety Severity | Depression Severity |
|---|---|---|---|---|---|
| | Model1 | Model2 | Model3 | Model4 | Model5 |
| Number of Hasslers | 0.139**<br>(0.0338) | 0.234**<br>(0.0349) | 0.133**<br>(0.0340) | 4.691**<br>(0.761) | 5.532**<br>(0.848) |
| Observations | 2343 | 2342 | 2343 | 2288 | 2288 |
| Individual controls | V | V | V | V | V |
| Network size FE | V | V | V | V | V |
| Outcome | Epigenetic inflammation score | Multi-morbidity | Waist to Hip Ratio | BMI | Height |
| | Model6 | Model7 | Model8 | Model9 | Model10 |
| Number of Hasslers | 0.00113**<br>(0.000315) | 0.130**<br>(0.0480) | 0.0115**<br>(0.00314) | 1.242**<br>(0.269) | -0.365<br>(0.282) |
| Observations | 2345 | 2158 | 1911 | 1967 | 1983 |
| Individual controls | V | V | V | V | V |
| Network size FE | V | V | V | V | V |

Note. Models 1–5 assess self-reported general health, mental health, physical health, anxiety severity, and depression severity, while Models 6–10 analyze objective biomarkers and anthropometric indicators, including log-transformed C-reactive protein (CRP), multimorbidity, waist-to-hip ratio, body mass index (BMI), and height. Coefficients represent the estimated effects of negative social ties on each outcome, with standard errors in parentheses. Higher values indicate worse health across all measures except height, which serves as a biologically stable control. Observations vary by model based on available data for each outcome. Statistical significance is denoted as follows: *p < .05, *$p < .01$ *(two-tailed tests).*

**Table S8. OLS regression models predicting self-reported health outcomes.**

| Outcome | General Health | Mental Health | Physical Health | Anxiety Severity | Depression Severity |
|---|---|---|---|---|---|
| Models | Model1 | Model2 | Model3 | Model4 | Model5 |
| Bath number (ref = 8615) | | | | | |
| 8732 | -0.0263 | -0.0263 | -0.0322 | -1.037 | 0.113 |
| | (0.0811) | (0.101) | (0.0982) | (1.617) | (1.684) |
| 9054 | -0.00272 | 0.0178 | -0.00889 | -0.407 | 0.335 |
| | (0.0874) | (0.103) | (0.0982) | (1.702) | (1.632) |
| 9109 | 0.131+ | 0.146 | 0.191* | 0.749 | 1.636 |
| | (0.0756) | (0.103) | (0.0831) | (1.813) | (2.007) |
| 9213 | 0.0107 | -0.00622 | -0.0320 | 4.397* | 2.766 |
| | (0.109) | (0.0966) | (0.122) | (1.905) | (1.763) |
| 11277 | -0.256 | -0.153 | -0.108 | -7.606* | -10.32** |
| | (0.263) | (0.239) | (0.291) | (2.876) | (3.775) |
| 13762 | -0.0883 | -0.0675 | 0.00121 | -7.729** | -5.609* |
| | (0.125) | (0.108) | (0.129) | (1.761) | (2.523) |
| % Leukocyte cells | -0.473** | -0.386** | -0.491** | -7.698** | -8.040** |
| | (0.135) | (0.141) | (0.128) | (2.455) | (2.644) |
| Age | -0.000317 | -0.00961** | 0.00256 | -0.203** | -0.165** |
| | (0.00160) | (0.00170) | (0.00166) | (0.0308) | (0.0329) |
| Race (ref = White) | | | | | |
| Black | 0.0992 | -0.160+ | 0.0163 | -3.137+ | -5.615** |
| | (0.0854) | (0.0923) | (0.0761) | (1.642) | (1.794) |
| Other | 0.107 | -0.0205 | 0.130 | 1.041 | -2.029 |
| | (0.0979) | (0.109) | (0.136) | (1.710) | (1.914) |
| FEMALE | 0.122* | 0.240** | 0.219** | 3.501** | 3.468** |
| | (0.0569) | (0.0525) | (0.0458) | (0.975) | (0.961) |
| Education (ref = HS or less than HS) | | | | | |
| Some college | -0.223** | -0.258** | -0.250** | -0.872 | 0.149 |
| | (0.0610) | (0.0823) | (0.0562) | (1.640) | (1.324) |
| College or higher | -0.402** | -0.393** | -0.471** | -1.882 | -1.505 |
| | (0.0626) | (0.0831) | (0.0645) | (1.866) | (1.433) |
| Martial Status (ref = Never-married) | | | | | |
| Married or living with a partner | -0.148 | -0.0802 | -0.184* | -2.196 | -3.476* |
| | (0.0938) | (0.0944) | (0.0865) | (1.795) | (1.686) |
| Widowed/Divorced/Separated | 0.0228 | 0.145 | -0.0555 | 1.622 | 1.960 |
| | (0.113) | (0.126) | (0.105) | (2.096) | (2.090) |
| Number of hasslers | 0.139** | 0.234** | 0.133** | 4.691** | 5.532** |
| | (0.0338) | (0.0349) | (0.0340) | (0.761) | (0.848) |
| Constant | 3.019** | 3.396** | 2.807** | 37.78** | 45.71** |
| | (0.362) | (0.302) | (0.403) | (7.286) | (8.549) |
| Observations | 2343 | 2342 | 2343 | 2288 | 2288 |

Note. All models control for network size dummies. Standard errors are in parentheses (+ p < 0.1, * p < 0.05, ** p < 0.01).

**Table S9. OLS regression models predicting objective health outcomes.**

| Outcome | Epigenetic inflammation score | Multi-morbidity | Waist to Hip Ratio | BMI | Height |
|---|---|---|---|---|---|
| Models | Model6 | Model7 | Model8 | Model9 | Model10 |
| Bath number (ref = 8615) | | | | | |
| 8732 | -0.00325** | 0.0113 | -0.00325 | -0.979 | -0.872 |
| | (0.000898) | (0.129) | (0.0114) | (0.917) | (1.412) |
| 9054 | -0.00337** | 0.00433 | -0.00680 | -1.018 | 0.768 |
| | (0.000831) | (0.0941) | (0.0118) | (0.998) | (1.167) |
| 9109 | -0.00216* | 0.0997 | -0.00355 | -1.055 | 0.381 |
| | (0.000868) | (0.122) | (0.00893) | (0.738) | (1.569) |
| 9213 | -0.00379** | -0.0450 | -0.00655 | -2.455** | -0.0257 |
| | (0.000932) | (0.115) | (0.0106) | (0.914) | (1.173) |
| 11277 | 0.00152 | -0.253 | 0.0221 | 0.975 | -0.521 |
| | (0.00226) | (0.299) | (0.0162) | (1.987) | (1.233) |
| 13762 | -0.00476** | 0.159 | -0.00665 | 0.772 | 1.230 |
| | (0.00168) | (0.181) | (0.0136) | (1.361) | (2.058) |
| % Leukocyte cells | -0.146** | -0.512+ | 0.0104 | 2.148* | -3.534* |
| | (0.00177) | (0.295) | (0.0107) | (1.074) | (1.371) |
| Age | 0.000143** | 0.0267** | 0.000860** | 0.000594 | -0.104** |
| | (0.0000189) | (0.00229) | (0.000283) | (0.0168) | (0.0143) |
| Race (ref = White) | | | | | |
| Black | 0.00380** | 0.0377 | 0.0105 | 1.223 | -1.096 |
| | (0.00115) | (0.115) | (0.00907) | (0.792) | (0.796) |
| Other | 0.00248* | -0.0188 | 0.00643 | 1.169 | -2.549** |
| | (0.000989) | (0.101) | (0.0125) | (0.968) | (0.777) |
| FEMALE | 0.00241** | 0.112 | -0.0789** | 0.674 | -14.52** |
| | (0.000448) | (0.0743) | (0.00553) | (0.496) | (0.578) |
| Education (ref = HS or less than HS) | | | | | |
| Some college | -0.00137+ | -0.190+ | -0.00771 | 0.00161 | 0.482 |
| | (0.000795) | (0.112) | (0.00838) | (0.845) | (0.408) |
| College or higher | -0.00474** | -0.203+ | -0.0163** | -1.304+ | 1.624* |
| | (0.000936) | (0.103) | (0.00558) | (0.657) | (0.760) |
| Martial Status (ref = Never-married) | | | | | |
| Married or living with a partner | -0.000308 | -0.0125 | 0.0309** | 0.992 | -0.423 |
| | (0.000943) | (0.107) | (0.0109) | (0.948) | (0.793) |
| Widowed/Divorced/Separated | 0.00299** | -0.0120 | 0.0297* | 1.236 | -0.870 |
| | (0.000964) | (0.123) | (0.0126) | (1.233) | (0.886) |
| Number of hasslers | 0.00113** | 0.130** | 0.0115** | 1.242** | -0.365 |
| | (0.000315) | (0.0480) | (0.00314) | (0.269) | (0.282) |
| Constant | 0.155** | 0.0837 | 0.909** | 25.51** | 186.2** |
| | (0.00363) | (0.358) | (0.0307) | (2.515) | (3.916) |
| Observations | 2345 | 2158 | 1911 | 1967 | 1983 |

Note. All models control for network size dummies. Standard errors are in parentheses (+ $p < 0.1$, * $p < 0.05$, ** $p < 0.01$).

**Table S10. Sensitivity Analysis of The Impact of Negative Social Ties on Epigenetic Aging Clocks**

| | PACE | | | | | | | |
|---|---|---|---|---|---|---|---|---|
| | Model1 | Model2 | Model3 | Model4 | Model5 | Model6 | Model7 | Model8 |
| Number of Hasslers | 0.0152** | 0.0149** | 0.0138** | 0.0130** | 0.0150** | 0.0138** | 0.0109** | 0.0118** |
| | (0.00384) | (0.00390) | (0.00413) | (0.00404) | (0.00386) | (0.00366) | (0.00341) | (0.00412) |
| Observations | 2345 | 2339 | 1987 | 2345 | 2294 | 2345 | 2341 | 2345 |
| | AgeAccelGrim2 | | | | | | | |
| | Model9 | Model10 | Model11 | Model12 | Model13 | Model14 | Model15 | Model16 |
| Number of Hasslers | 0.784** | 0.771** | 0.810** | 0.738** | 0.787** | 0.739** | 0.496** | 0.654** |
| | (0.189) | (0.182) | (0.203) | (0.198) | (0.187) | (0.170) | (0.138) | (0.198) |
| Observations | 2345 | 2339 | 1987 | 2345 | 2294 | 2345 | 2341 | 2345 |
| Controls | | | | | | | | |
| Individual demographics | V | V | V | V | V | V | V | V |
| Network size FE | V | V | V | V | V | V | V | V |
| COVID-19 period | | V | | | | | | |
| Health Insurance | | V | | | | | | |
| Prior Charlson index | | | V | | | | | |
| Medical visit (past 3yr) | | | V | | | | | |
| Life-time multi-morbidity | | | | V | | | | |
| Your importance to others | | | | | V | | | |
| Others' dependence on you | | | | | V | | | |
| Occupation | | | | | | V | | |
| Smoking Status | | | | | | | V | |
| ACEs | | | | | | | | V |

Note. Models 1–8 estimate associations with PACE, and Models 9–16 estimate associations with AgeAccelGrim2. Coefficients represent the effects of the number of hasslers on each outcome, with standard errors in parentheses. All models adjust for demographic characteristics (age, gender, race/ethnicity, education, and marital status) and include additional robustness controls for survey period (COVID-19 exposure), health insurance status, Charlson multimorbidity index (from respondents' EHR records over the past 3 years), lifetime multi-morbidity index from the survey, psychosocial attitudinal measures, occupation, and smoking status (a known epigenetic confounder). Network size fixed effects are included to account for differences in the number of alters, ensuring that estimates are not confounded by absolute network size. Statistical significance: * $p < .05$, ** $p < .01$ (two-tailed tests).

**Table S11. Longitudinal analyses of the association between negative social ties and self-reported health outcomes.**

| | General Health (T=1) | | Mental Health (T=1) | | Physical Health (T=1) | |
|---|---|---|---|---|---|---|
| | Model1 | Model2 | Model3 | Model4 | Model5 | Model6 |
| Number of Hasslers | 0.145** | 0.106+ | 0.157** | 0.0859* | 0.229** | 0.131* |
| | (0.0438) | (0.0556) | (0.0435) | (0.0335) | (0.0687) | (0.0579) |
| Observations | 1271 | 1271 | 1271 | 1270 | 1271 | 1271 |
| Controls | | | | | | |
| Individual controls | V | V | V | V | V | V |
| Network size dummies | V | V | V | V | V | V |
| Baseline General Health | | V | | | | |
| Baseline Mental Health | | | | V | | |
| Baseline Physical Health | | | | | | V |

Note. Estimates reflect average marginal effects from longitudinal models predicting self-reported general, mental, and physical health outcomes in follow-up survey data collected between June 2022 and September 2022. Each model includes the same lagged dependent variable used in the corresponding baseline specification to account for prior self-reported health. Individual controls include age, gender, race/ethnicity, education, and marital status. Network size dummies account for differences in social network size, ensuring that the relative impact of negative ties is not confounded by absolute network size. Statistical significance is denoted as follows: + p < .10, * p < .05, ** p < .01 (two-tailed tests).

**Table S12. Interaction models testing whether marital status and the COVID-19 period moderate the association between the number of hasslers and biological aging.**

| | PACE | | | AgeAccelGrim2 | | |
|---|---|---|---|---|---|---|
| Period | All | COVID | Non-COVID | All | COVID | Non-COVID |
| The AME for each subgroup | | | | | | |
| Never-married | 0.0250** | 0.0587** | 0.0178+ | 0.901** | 1.938** | 0.674+ |
| | (0.00918) | (0.0146) | (0.00989) | (0.327) | (0.549) | (0.353) |
| Married or living with a partner | 0.0112* | 0.00951 | 0.0135* | 0.747* | 1.156* | 0.716* |
| | (0.00442) | (0.00943) | (0.00565) | (0.283) | (0.445) | (0.292) |
| Widowed/Divorced/Separated | 0.0172** | -0.00651 | 0.0330** | 0.768* | 0.230 | 1.083** |
| | (0.00624) | (0.0163) | (0.00827) | (0.354) | (0.470) | (0.399) |
| Observations | 2345 | 915 | 1430 | 2345 | 915 | 1430 |

**Note.** Full interaction models were estimated to assess whether spousal/partner dynamics or the COVID-19 context account for the observed association between hassling ties and biological aging. Models include interactions between marital status (married/living with a partner; widowed/divorced/separated; never married), number of hasslers, and COVID versus pre-COVID period for both PACE and GrimAge2, in addition to baseline controls (age, gender, race/ethnicity, education, marital status, network size dummies). Statistical significance is denoted as follows: + p < .10, * p < .05, ** p < .01 (two-tailed tests).

**Table S13. Gender-stratified models testing whether the association between the number of hasslers and biological aging differs for men and women (PACE and AgeAccelGrim2).**

| | PACE | | AgeAccelGrim2 | |
|---|---|---|---|---|
| Gender Subgroup | Male | Female | Male | Female |
| Number of Hasslers | 0.00372 | 0.0199** | 0.805** | 0.674** |
| | (0.00659) | (0.00573) | (0.269) | (0.177) |
| Observations | 912 | 1433 | 912 | 1433 |

**Note.** Coefficients represent the estimated effects of negative social ties on biological aging outcomes, with standard errors in parentheses. Individual controls include age, gender, race/ethnicity, education, and marital status. Network size dummies account for differences in social network size, ensuring that the relative impact of negative ties is not confounded by absolute network size. Statistical significance is denoted as follows: + $p < .10$, * $p < .05$, ** $p < .01$ (two-tailed tests).

**Table S14. Sensitivity of associations between the number of hasslers and epigenetic aging outcomes to unmeasured confounding from *konfound* analysis.**

| | PACE | AgeAccelGrim2 |
|---|---|---|
| **RIR cases** | **1132 (48.3%)** | **1010 (43.1%)** |
| threshold effect size | 0.006 | 0.345 |
| **Conditional ITCV for the number of hasslers** | **0.039** | **0.032** |
| Threshold correlation (confounder, outcome) | 0.198 | 0.179 |
| Threshold correlation (confounder, predictor) | 0.198 | 0.179 |
| Conditional ITCV for other covariates | | |
| Batch 2 (vs Batch 1) | 0.001 | 0.000 |
| Batch 3 (vs Batch 1) | 0.001 | 0.000 |
| Batch 4 (vs Batch 1) | 0.000 | 0.000 |
| Batch 5 (vs Batch 1) | 0.001 | 0.000 |
| Batch 6 (vs Batch 1) | 0.001 | 0.002 |
| Batch 7 (vs Batch 1) | 0.002 | 0.001 |
| Leukocytes | 0.034 | 0.037 |
| Age | -0.003 | 0.030 |
| Race : Black (Vs White) | 0.004 | 0.003 |
| Race : Other (Vs White) | 0.001 | 0.001 |
| Female (vs Male) | 0.006 | -0.007 |
| Educ: Some college (vs HS or less than HS) | 0.001 | 0.001 |
| Educ: College or higher (vs HS or less than HS) | 0.000 | 0.000 |
| Marital Status: Married or Cohabited (vs never-married) | 0.001 | 0.002 |
| Marital Status: Widowed/Divorced/Separated (vs never-married) | 0.003 | 0.006 |

Note. Values represent robustness statistics from *konfound* analyses evaluating the extent of unmeasured confounding required to eliminate the observed associations between the number of hasslers and two epigenetic aging measures (PACE and AgeAccelGrim2). RIR (Robustness of Inference to Replacement) indicates the percentage and number of cases that would need to be replaced with counterfactual cases showing null effects to render the association nonsignificant at $p < 0.05$. ITCV (Impact Threshold for a Confounding Variable) reflects the minimum product of correlations that an omitted confounder would need with both the predictor and the outcome to explain away the effect; corresponding threshold correlations are shown. Conditional ITCV values for all measured covariates illustrate that none approach the threshold required to overturn the focal association.

**Table S15. Associations Between Hasslers and Biological Aging Across Age Groups.**

| Outcomes | | | Pace | | | |
|---|---|---|---|---|---|---|
| Age Group | 18-30 | 31-40 | 41-50 | 51-60 | 61-70 | 71+ |
| N of Hasslers | 0.0357** | 0.00105 | 0.0157 | -0.000476 | 0.00370 | 0.00911 |
| | (0.00891) | (0.00767) | (0.0166) | (0.00740) | (0.00807) | (0.0127) |
| Observations | 375 | 425 | 339 | 370 | 407 | 363 |
| Outcomes | | | AgeAccelGrim2 | | | |
| Age Group | 18-30 | 31-40 | 41-50 | 51-60 | 61-70 | 71+ |
| N of Hasslers | 0.948** | 0.340 | 0.590+ | 1.168** | 0.0780 | 0.758+ |
| | (0.290) | (0.282) | (0.316) | (0.234) | (0.269) | (0.432) |
| Observations | 375 | 425 | 339 | 370 | 407 | 363 |

Note. Coefficients represent the average marginal effects of negative social ties on biological aging outcomes, with standard errors in parentheses. Individual controls include age, gender, race/ethnicity, education, and marital status. Network size dummies account for differences in social network size, ensuring that the relative impact of negative ties is not confounded by absolute network size. Statistical significance is denoted as follows: + p < .10, * p < .05, ** p < .01 (two-tailed tests).
